# Active Anti-erosion Protection Strategy in Tamarisk (*Tamarix aphylla*)

**DOI:** 10.1038/srep03429

**Published:** 2013-12-05

**Authors:** Zhiwu Han, Wei Yin, Junqiu Zhang, Shichao Niu, Luquan Ren

**Affiliations:** 1 Key Laboratory of Bionic Engineering, Ministry of Education, Jilin University, Changchun 130022, P. R. China

## Abstract

Plants have numerous active protection strategies for adapting to complex and severe environments. These strategies provide endless inspiration for extending the service life of materials and machines. Tamarisk (*Tamarix aphylla*), a tree that thrives in raging sandstorm regions, has adapted to blustery conditions by evolving extremely effective and robust erosion resistant characteristics. However, the relationships among its surface cracks, internal histology and biomechanics, such as cracks, rings, cells, elasticity modulus and growth stress, which account for its erosion resistance, remain unclear. This present study reveals that the directionally eccentric growth rings of tamarisk, which are attributed to reduced stress and accelerated cell division, promote the formation of surface cracks. The windward rings are more extensive than the leeward side rings. The windward surfaces are more prone to cracks, which improves erosion resistance. Our data provide insight into the active protection strategy of the tamarisk against wind–sand erosion.

Plants survive adversity by integrating growth responses to diverse environmental signals^1^,^2^,^3^. Active protection strategies for overcoming adversity offer more opportunities for improving the service life of materials and machines^4^. Active protection strategies commonly implement multiple mechanisms simultaneously, such as in maize^5^, nicotiana^6^, arabidopsis^7^, acacia^8^ and cotton^9^. Generally, most deserts have strong windy conditions, but tamarisk (*Tamarix aphylla*), which has adapted to such blustery conditions, suffer only minor scratches, thereby proving that it has developed high erosion resistance.

The surface cracks of trees, ring eccentricity and biomechanical properties are the most common physical phenomena observed during trees growth. In many cases, the surface cracks are normal physical phenomenon when the trees grow. Tree ring asymmetry develops during growth as an adaptive response to changes in loading^10^. Trees are sessile organisms that are susceptible to environmental fluctuation. During tree growth, the branches need to adapt to increasing gravitational^11^ or windy^12^,^13^ loads that bend the branch downwards. Furthermore, trees growing on slopes transformed by mass movements are tilted^14^. Given that more materials are added to the lower side of branches, the cross-section of the branches show rings with pronounced radial eccentricity^15^,^16^. The biomechanical properties of trees are extremely complex because these organisms change their size and shape and the material properties of their stems as they grow in size and height^17^. A study on the relationships between growth stress and several morphological parameters in Beech (*Fagus sylvatica L.*) showed that, in the case of a small inclination, growth stress is as close to expected to the biomechanics of restoring verticality. In comparison, trees that exhibit a larger inclination probably evolve a different mechanical solution: a rather large crown, lower tree slenderness, and a sufficient asymmetry in growth stress to prevent a high inclination because of growth^18^. However, the relationships among the surface morphological cracks, internal structure, and biomechanics of the tamarisk have not been investigated.

This study investigated the relationships among surface cracks, eccentric structure, and biomechanics of the tamarisk to understand its active anti-erosion protection strategy. Surface crack is one of the characteristics of the tamarisk which enables it to adapt to the wind–sand erosion. The emergence of surface cracks reduces surface growth stress and promotes cell division and results in eccentric growth, which, in turn, promotes the cracks.

## Results

### Erosion test in the windward and leeward sides of tamarisk surface

**Figure 1:**
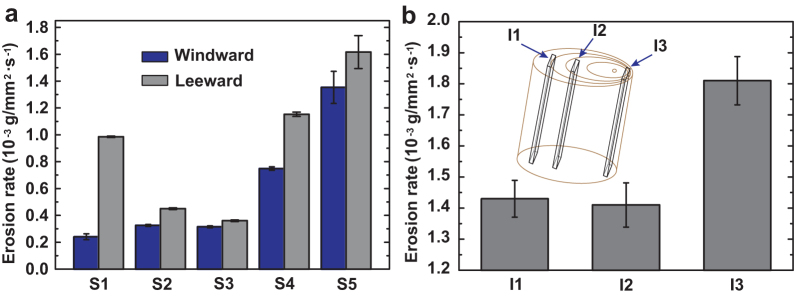
Erosion rates of the windward and leeward side surfaces of samples with different diameters and internal different positions. (a) (*S1–S2*) The diameters were approximately 8, 14, 24, 30, and 44 mm, respectively. (b) (*I1–I3*) The internal different positions with diameter 14 mm (*S2*).

To analyze the effect of wind and sand on the growth of tamarisk, the surface and internal erosion of tamarisk in the windward and leeward sides were compared. Figure 1a shows the surface erosion rates of the samples with different diameters after reaching their steady state. The erosion rates on the windward side were much lower than those on the leeward side of the tamarisk surface with different diameters. This result indicates that the windward side of tamarisk trees have greater erosion resistance against sandstorms. To study the erosion without the surface morphology, the erosion rates of the different internal positions of the tamarisk with a 14 mm diameter (*S2* in Fig. 1a) were tested, as shown in Fig. 1b. The erosion rate of the transition zone (I2) was lowest and the erosion rate of the windward side (I1 zone) was considerably lower than that of the leeward side (I3 zone).

### Surface crack and cross-sectional structure of tamarisk

**Figure 2:**
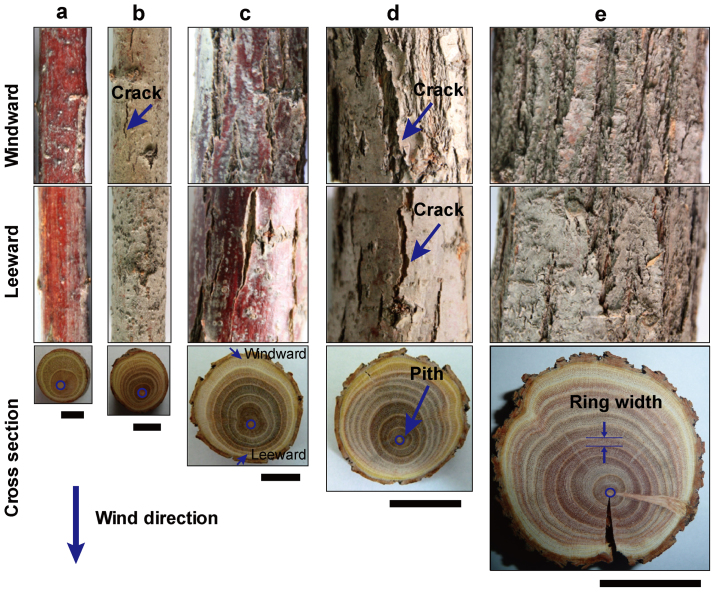
Surface crack and asymmetric radial cross-section. Panels (a)–(e) show the surface cracks of the windward and leeward side and the corresponding cross-sectional microscopic structures. The diameters of the samples were approximately 8, 14, 24, 30, and 44 mm, respectively. (a) Cracks were absent in both the windward side and the leeward side. (b) Cracks started to appear in the windward, but not in the leeward side. The number and size of the cracks gradually increased with increasing trunk diameter. The cracks in the windward side were larger and more numerous than those in the leeward side of the same trunk, respectively (c–e). The piths of tamarisk with different diameters were far away from wind direction, or windward side (bottom, a–e). Scale bars, 4 mm (a); 7 mm (b); 8 mm (c); 15 mm (d), and 22 mm (e).

The appearance and structure of tamarisk trunks were the result of environmental stimulation. Figure 2 shows the surface cracks of the windward and leeward sides of the tamarisk trunks and the corresponding cross-sectional microscopic structures. Cracks were absent in both the windward and the leeward sides in the trunks with smaller diameters, as shown in the top and middle panels of Fig. 2a. The cracks in the windward appeared on the trunk that grew to a certain size (top panel in Fig. 2b), but the cracks on the leeward side were absent (middle panel in Fig. 2b). When the trunks grew to sufficient sizes, the quantity and size of the cracks in the windward side were greater and larger than those on the leeward side of the same position of the same trunk, as shown in the top and middle panels of Figs. 2c–2e.

The cross sections of the trunks with different diameters were eccentrically shaped, as shown in the bottom panel of Figs. 2a–2e. Tamarisk subjected to stimulation during growth developed narrower rings, producing very thin rings during dry years and thick rings during wet years. The eccentric growth of tamarisk was related to environmental stimulation. The pith or centre was far away from the windward side. The rings in the windward side were significantly wider than those in the opposite side. The wider rings exhibited the same orientation, with multiple cracks.

**Figure 3:**
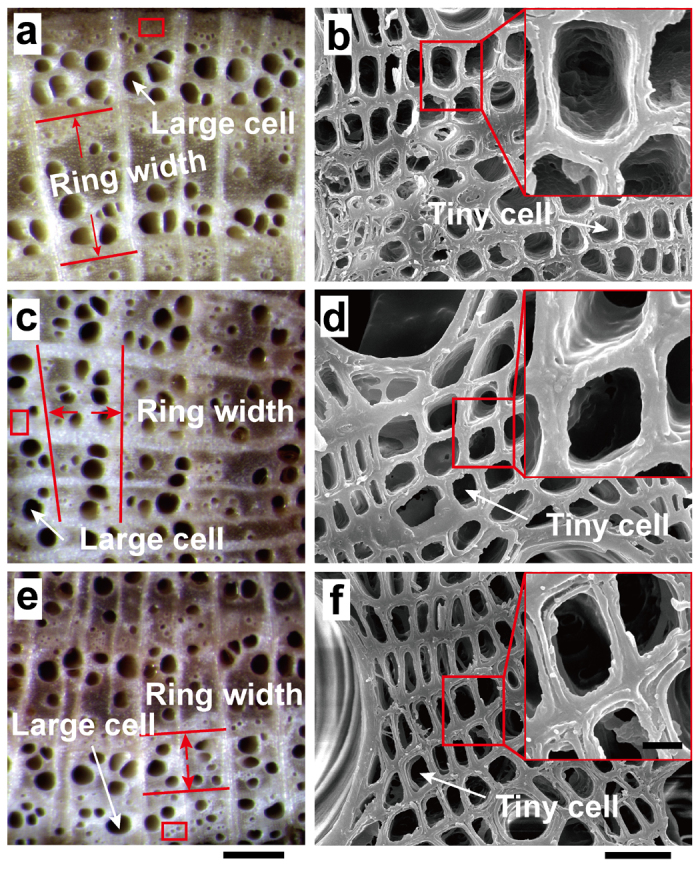
Anatomic observation of the cross section of samples in Fig. 2b. (a) Transverse section optical micrograph of tissues from the windward side. The red box was magnified (b) through scanning electron microscopy (SEM). (c) Transverse section optical micrograph of tissues from the transition zone between the windward side and the leeward side. The red box was magnified (d) through SEM. (e) Transverse section optical micrograph of the tissues from the leeward side within the same ring as the windward side specimen. The red box was magnified (f) through SEM. The insets on panels (b), (d), and (f) show highly magnified SEM micrographs of tiny vascular cells. The cell wall thickness remained unchanged. Ring width gradually deceased from the windward side to the leeward side. Scale bars, 500 μm (a), (c), and (e); 20 μm (b), (d), and (f); and 5 μm in the insets.

Anatomical variations in the same growth ring were observed and investigated under a microscope. The cross-sectional cell structure in the windward side, the transition zone between the windward side and the leeward side, and the leeward side of the same ring are shown in Figs. 3a, 3b; 3c, 3d; and 3e, 3f, respectively. The transverse section reveals large vascular cells in the same ring were almost the same size between the windward side and the leeward side (Figs. 3a, 3c, and 3e). However, the large vascular cells in the windward side were healthier than those in the leeward side. The changes in cell number within the tissue directly demonstrated cell division. The partially enlarged photos show that the tiny vascular cells were uneven, round, oval, and strip (Figs. 3b, 3d and 3f). However, the sizes of the tiny vascular cell were similar between the windward side and the leeward side. Histologic observations indicated that the eccentric growth was dominated by cell division.

### Ring width and biomechanics in the cross sections

**Figure 4:**
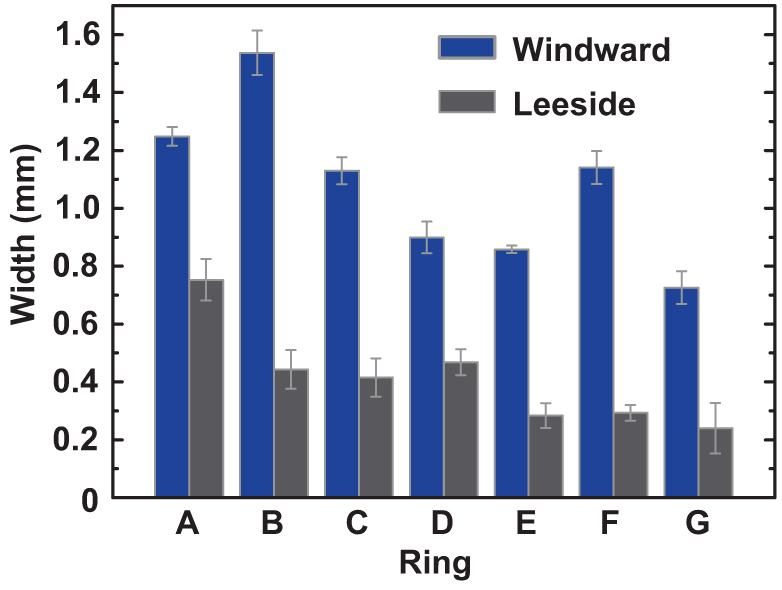
Characteristics of the tamarisk cross section in Fig. 2b. Ring width of tamarisk in the windward and leeward sides. (*A*–*G*) Rings from the pith to the edges.

The rings in the windward side were wider than their corresponding continuation in the leeward side (Fig. 4). The width of the windward side was about twice that of the leeward side. The ring widths of the windward or leeward side were different because the rings were affected differently by annual environmental conditions. Different years were subject to different environmental effects in the windward or leeward side.

**Figure 5:**
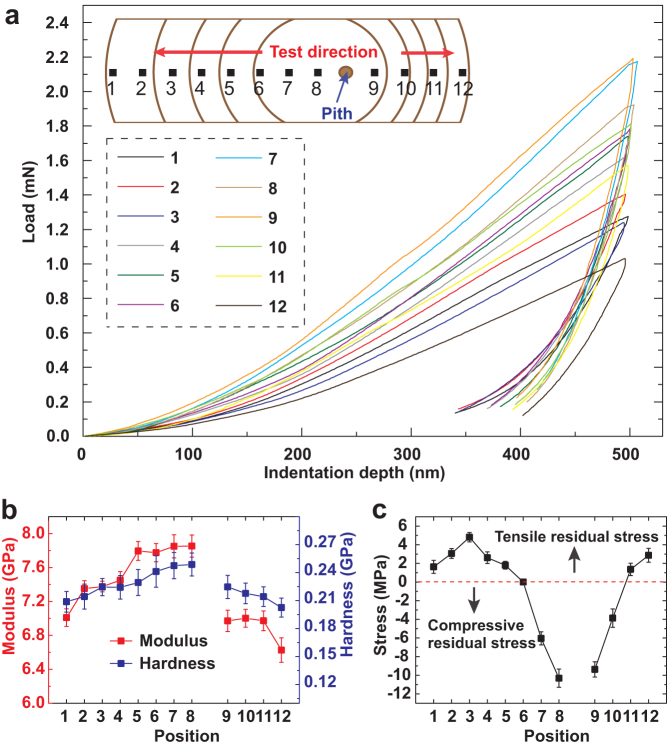
Biomechanics of the tamarisk cross section in Fig. 2b. (a) Representative nanoindentation load–displacement curves at 500 nm indentation depth. The inset shows the positions of indentation. Nanoindentation was tested from the pith to the edges on both sides. The distance between the two points was 1 mm. Each point was tested 10 times on nearby areas to calculate the standard deviation. (b) Modulus and hardness of the different positions. The modulus and hardness of the windward side were higher than those of the leeward side. (c) Distribution of residual stress from the windward side to the leeward side. The red line represents stress-free. The top and bottom zones denote the tensile residual stress and the compressive residual stress zone, respectively. Residual stress in the stem with tension compression forces was equilibrium. The edge of the tamarisk trunk was subjected to tensile stress, and the inner part was subjected to compressive stress.

Nanoindentation was used to study the biomechanics, modulus, hardness and stress of the tamarisk and understand the relationships among its surface morphology, internal structure, and biomechanics. Figure 5a shows experimentally determined load–displacement curves obtained for indentations at a maximum depth of 500 nm. Compared to the stress-free loading curve, the figure shows that the load required to penetrate up to 500 nm increases for compressive residual stress while an opposite effect is obtained for tensile residual stress, that is, the load decreases. The shape of the loading curve had changed correspondingly. Figure 5b shows the modulus and hardness obtained from the nanoindentation experiments. Modulus and hardness increased from edges to pith, from points *1* to *8* and from *12* to *9*. Average modulus and hardness of the windward side were higher than those of the leeward side. Figure 5c shows the residual stress variation in the tamarisk cross section. The origins of growth stress have been extensively analyzed. The maturation of cells and meristem caused growth stress^19^,^20^. During maturation, the newly formed cells that grow on the stem periphery every year, contracted longitudinally, whereas the already formed lignified cells impeded this contraction. This resistance to contraction caused tension, which contributed to the protection of new cells from bruising. The stress within the tree tissues attained equilibrium^21^. In accordance with the theory by Kübler^22^, growth stress was highest at the stem periphery. A tension zone formed closest to the bark and a compression zone formed nearest the centre of the tamarisk trunk, resulting in a tension-free line (red line of Fig. 5c) between the two zones. However, the stress on the windward side initially (from points *1* to *3*) increased and then (from points *3* to *8*) decreased. The maximum tension stress occurred at the subsurface on the windward side.

## Discussion

Surface erosion caused by particles during the flow wear of particulates was reduced by the crack on the surface. Studies on the surface morphology of the carapace of the desert scorpion^23^,^24^ and the non-smooth surface morphology of ribs^25^,^26^ showed that the grooved surface has better erosion resistance than the non-grooved one. Grooves can enhance fluid turbulence, which leads to a change of the flow field around the groove surface and, subsequently, a change of the particle motion pattern. Some particles leave the surface along with air flow without impact, and these particles impact the surface if the surface is smooth. Therefore, the number of particles impacting the surface decreases. As a result of the decrease in flow velocity, the velocity of particles in the two-phase flow decreases as well. Rotating flow in the groove can absorb particle energy that is used for impacting and correspondingly reduces the energy used in impact. Based on the above mechanism of erosion resistance of the grooved surface, the grooved pattern, that is, the cracks on the surface of the tamarisk body, clearly contributes to erosion resistance.

According to the micro-cutting theory of Finnie^27^,^28^, the erosion weight loss of the ductile material is directly proportional to the elastic modulus. Therefore, the windward side (internal, excluding bark) has better anti-erosion than the leeward side because the elastic modulus of the windward side is larger than that of the leeward side (Fig. 1b). High elastic modulus is another important reason for the erosion resistance in the tamarisk.

**Figure 6:**
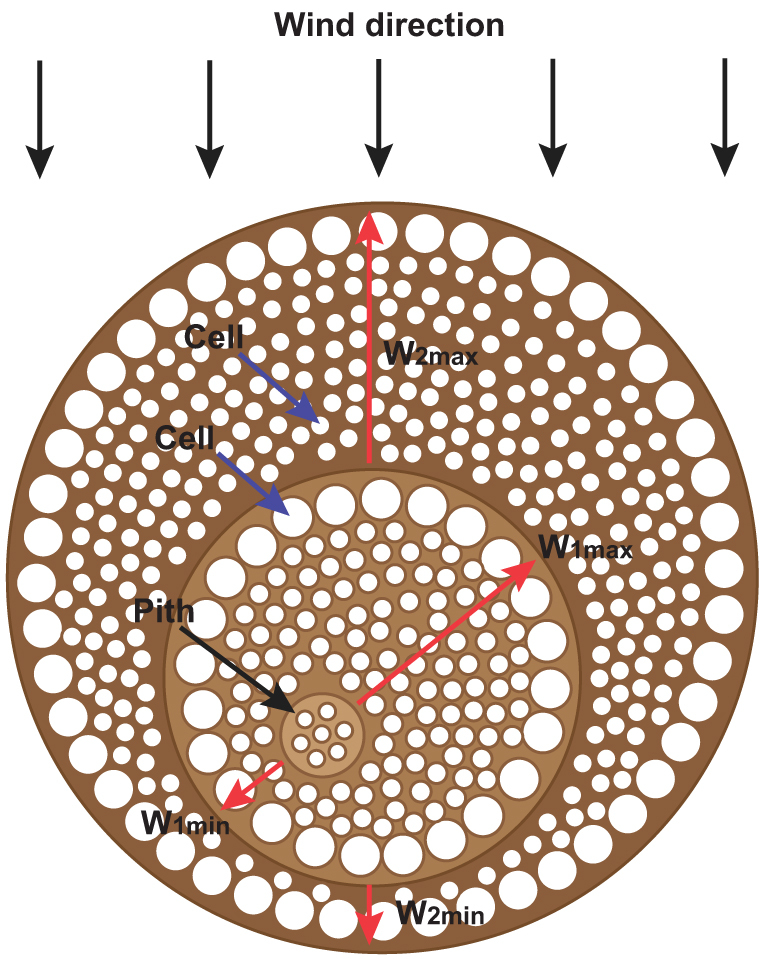
Relationships among wind–sand erosion, eccentric growth, and tissue growth. Red arrows mark the position of maximum ring width. *W*_1max_ and *W*_1min_ are the widest and the narrowest of the first ring, respectively; *W*_2max_ and *W*_2min_ are the widest and the narrowest of the second ring, respectively. The directions of *W*_1max_ and *W*_2max_ are different. Blue arrows mark the vascular cells. The sizes of the large or tiny vascular cells in the windward and leeward sides are the same.

The tamarisk has high wind–sand erosion resistance. Meanwhile, wind–sand erosion affects the internal directional growth (eccentricity) of the tamarisk. In addition, the eccentric growth is caused by the fast cell division. Eccentric growth of trees is affected by external conditions, such as snow pressure, competition of adjacent trees, and topography, et al^29^,^30^. In this study, vertical tamarisks were collected on a flat terrain to eliminate the influence of these factors. The eccentric growth of the tamarisk was observed for the entire cross-section or all the rings. However, the position of the maximum ring width does not always move in one particular direction. It appears to be distributed randomly in the cross-section (Fig. 6), though the directions are directed at the windward side. Wind–sand erosion occurring at exactly the same direction every time is impossible. In other words, wind–sand erosion does not occur at the same position of the tamarisk every time. Therefore, a deviation phenomenon occurs, such that the maximum width directions of the rings are different. We can speculate that the eccentric growth is caused by wind–sand erosion, which is well verified in the bottom panels of Fig. 2a. The sizes of the large or tiny vascular cell are the same in the windward and leeward sides. The internal asymmetric growth of the tamarisk is due to the quantity of large and small vascular cells. The number of cells in the windward side is greater than that in the leeward side. The number of cells is associated with the cell division rate; that is, the higher the cell division rate, the greater the number of cells. The directionality growth of the tamarisk is caused by fast cell division. That is, the eccentric growth of organization is caused by fast cell division.

**Figure 7:**
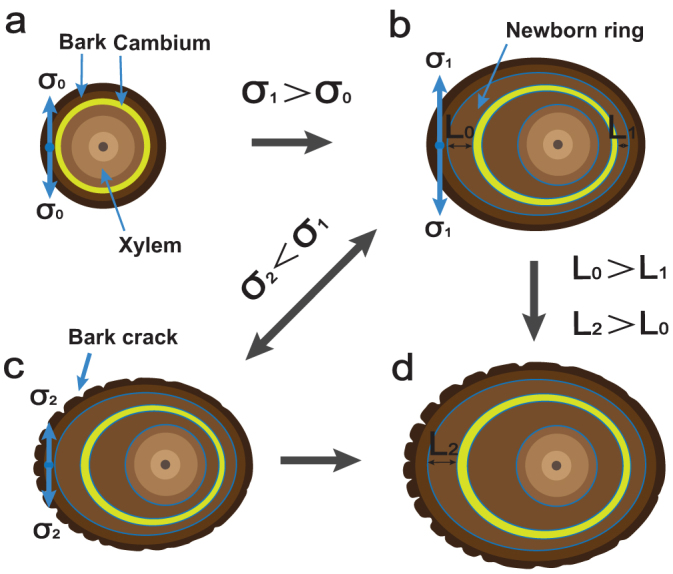
Schematic diagram of the relationships among surface cracks, ring eccentricity, and growth stress. (a) Tamarisk grows symmetrically with no erosion. The yellow circle represents the cambium. The outer and inner parts are the cambium and xylem, respectively. The initial near–surface tensile stress in the bark is σ_0_. (b) Tamarisk begins to grow after wind–sand erosion. The newborn ring on the windward side (L_0_) is thicker than that on the leeward side (L_1_). The near–surface tensile stress is σ_1_ after wind–sand erosion, and σ_0_ is greater than σ_1_. (c) The cracks are most likely towards the direction of the rapid growth. The surface tensile stress of the windward is reduced. The cambium has lower growth resistance; thus, its growth rate is higher. The surface stress is by the presence of cracks, that is, the surface stress σ_1_ is greater than σ_2_. (d) Growth and crack formation occur at the same time. The rings (L_2_) on the windward side are thicker than the newborn rings (L_0_).

Study the relationships among surface cracks, eccentric growth, and growth stress is important to understand the active protection strategy of the tamarisk. We proposed a potential mechanism to characterize the structure–function relationship in this self-adaptive process for wind–sand erosion. Figure 7 shows the relationships among surface cracks, ring eccentricity, and growth stress. Ring growth is symmetrical under normal growth conditions, as shown in Fig. 7a. The cell division rate of the cambium (the growth layer between the bark and the xylem) increases when tamarisk is under stimulation from the external environment^31^. It subsequently produces thicker rings^32^, and the surface growth stress simultaneously increases (Fig. 7b). The stimulated site grows the cracks under increasing growth stress (Fig. 7c). The cracks reduce the surface stress. The inner tissues are maintained under compression, but the outer tissues are under equivalent tension. In this case, growth anisotropy is dictated by the inner tissues, whereas the isotropic outer epidermal wall provides the major resistance to growth^33^. Therefore, the surface stress is released because of the emergence of scars, which benefits rapid tamarisk growth. The leeward side exhibits slower growth than the normal one because the windward side undergoes rapid growth, which absorbs the majority of nutrients. The tamarisk growth rings exhibits asymmetric growth, and the growth and crack form at the same time (Fig. 7d). Consequently, the external and internal stresses attain equilibrium. Cracks form earlier on the windward bark because of the rapid development of the new bark. The structural elements undergo controlled orientation, which leads to anisotropy that matches traction adaptation to environment directions^34^. This phenomenon also explains why the windward side stress initially increased and then decreased in Fig. 5c. The cracks promote stress reduction, in turn, promotes cell division. Cell division then promotes growth of eccentricity, which promotes the cracks. Specifically, the cracks in the windward side are larger and more numerous than those in the leeward side of the same trunk.

Our study explains that the tamarisk forms its surface morphology and elastic modulus with active protection itself through the use of wind–sand erosion, and verifies that wind–sand erosion promotes the growth of eccentric rings by changing growth stress. The emergence of surface cracks on the tamarisk reduces surface growth stress, which promotes fast cell division. A fast cell division results in internal eccentric growth, which directly affects the directionality of surface morphology and promotes the formation of surface cracks. The tamarisk improves erosion resistance by using surface cracks. These characteristics are valuable in designing materials surfaces because they effectively extend the lifetime of products^36^. Understanding of fundamental mechanism for growth processes in biological systems that respond and adapt to external mechanical stimuli is significant to the development of advanced functional surfaces^35^,^36^,^37^,^38^.

## Methods

### Sample collection

Tamarisk was collected in Baicheng City, Jilin Province, China (121°–124°22′ E; 44°13′–46°18′ N). The climate in Baicheng City is temperate continental monsoon in the Eurasian Centre. The average annual number of gale weather days is 24, and the average annual number of days with gale (less than grade 8) weather is 47. The annual average wind speed is up to 3.7 m/s^39^. The zonal soil types include gray desert soil, sandy clay loam, and sand soil. The annual average prevailing wind direction is south–southwest.

None of the trees completely grew vertically. Therefore, the tamarisk samples that leaned slightly (<5° at the base of the tamarisk), were collected to analyze the relationship between ring growth and wind–sand direction. The different trunks were approximately 8 mm to 44 mm in diameter.

### Erosion test

The erosion wear test system was designed to study erosion wear according to the American Society for Testing Materials (ASTM)–G76–83 standard^23^. The erosion test samples were 20 mm long. Three samples were tested for the same diameter trunk. The samples were initially eroded for 2 min to reach the steady–state erosion, which avoided adverse effects and ensured the accuracy of the test results. Erosion weight loss data were recorded every 10 s. Table 1 lists the erosion wear test conditions. Erosion rate was defined as the weight loss per unit time and per unit projected area (g/mm^2^·s^−1^).

### Nanoindentation experiments

Residual stress is defined as the stress that remains in a material without the application of an external load. Nanoindentation (Nano Indenter G200, Agilent Technologies Inc.) deforms materials at a very small scale and allows the determination of residual stress at the micro/nanoscale^40^,^41^. Nanoindentation was used to measure the residual stresses in tamarisk. The residual stress in the tamarisk cross sections were calculated using the load–displacement data obtained during indentation. During the nanoindentation tests, the Poisson's ratio was set to 0.45, the peak hold time was 20 s, the strain rate was 0.1 s^−1^, and the unload strain rate was 0.5 s^−1^.

The samples used for the nanoindentation test were obtained from areas on the tamarisk surface with no cracks. Before the tests, the tamarisk cross section were cleaned with ethanol (95%), dried at room temperature (25°C). Tamarisk sample was cut into 2 mm wide strip, as shown in the inset of Fig. 2a. Then it was inlaid into epoxy resin. The epoxy resin was cured at room temperature for 24 hours. The sample was cut and carefully polished into 2 mm thick sections. Given the porous surface, the imperforated or cellular region was considered to reflect the stress accurately and comprehensively.

Modulus, hardness, and residual stress were calculated from the measured indentation depth–load curve using the Oliver–Pharr and Suresh methods^42^,^43^,^44^. The calculation of residual stress required a stress-free point as a reference. The hardness of different positions of the tamarisk interior was different. Meanwhile, indentation depth was larger or smaller under the action of stress than under the stress-free state. The nanoindentation depth parameter was set to 500 nm in the test. Considering the hardness and indentation depth factors, the point where the hardness and indentation depth were near the average hardness and 500 nm respectively was defined as the stress-free reference point^45^. The following procedure is recommended to extract residual stresses, identify their sign (i.e., tensile or compressive), and identify the magnitude of residual stresses.

Indentation load decreases due to the release of compressive residual stress at a fixed penetration depth. Indentation load increases due to the release of tensile residual stress at a fixed penetration depth. The unloading curve can be fully described by the gradient of the initial part of the curve. Thus, the area of contact *A* can be obtained. 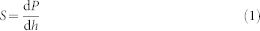

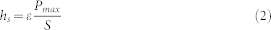
where *S* is defined as the slope of the upper portion of the unloading curve during the initial stages of unloading (also called the contact stiffness), *P*_max_ is the maximum load, *ε* is a constant that depends on the geometry of the indenter, *ε* = 0.75 for the Berkovich trigonal pyramid indenter, and *h*_s_ is the amount of sink-in. 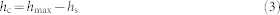
where *h*_c_ is the depth along which contact is made between the indenter and the specimen, and *h*_max_ is the maximum displacement. 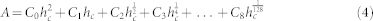
where *A* is the contact area for the surfaces. The coefficients for the area function are *C*_0_ = 24.65, *C_1_* = 202.7, *C_2_* = 0.03363, *C_3_* = 0.9318, *C_4_* = 0.02827, *C_5_* = 0.03716, *C_6_* = 1.763, *C_7_* = 0.04102, and *C_8_* = 1.881. 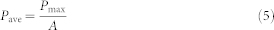
where *P*_ave_ is the average contact pressure.

For the tensile residual stress 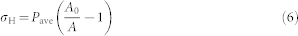
For the compressive residual stress 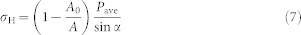
where *A*/*A*_0_ is the area ratio, with and without residual stresses, and *α* = 24.7° for the Berkovich trigonal pyramid indenter.

### Micrograph analysis

The transverse sections of the tamarisk stem were obtained using a hand sectioning method. The cross-section micrographs were observed under light microscopy (SteREO Discovery V12, Carl Zeiss). After gold coating, SEM studies were completed using a JSM–5600LV, JEOL.

## Author Contributions

Z.W.H. and L.Q.R. designed the project and guided the research. Z.W.H., W.Y. and J.Q.Z. carried out the experiments and wrote the paper. S.C.N. collected samples. All authors contributed to the discussions.

## Figures and Tables

**Table 1:** Erosion wear test conditions

Erosion particles	Particle diameter (mesh)	Pressure of air compressor (MPa)	Mass flow rate (g/s)	Impact angle (°)
Quartz sand	40–70	0.50–0.55	25	90

## References

[b1] Sun Y. D. *et al.* Structural basis for flg22–induced activation of the *Arabidopsis* FLS2–BAK1 immune complex. Science 342, 624–628 (2013). 24114786 10.1126/science.1243825

[b2] Bhushan B. & Jung Y. C. Natural and biomimetic artificial surfaces for superhydrophobicity, self–cleaning, low adhesion, and drag reduction. Prog. Mater Sci. 56, 1–108 (2011).

[b3] Ju J. *et al.* A multi–structural and multi–functional integrated fog collection system in cactus. Nat. Commun. 3, 1247 (2012). 23212376 10.1038/ncomms2253 PMC3535335

[b4] Nosonovsky M. & Bhushan B. Green tribology: principles, research areas and challenges. Philos. Trans. R. Soc. London, Ser. A 368, 4677–4694 (2010). 10.1098/rsta.2010.0200 20855315

[b5] Rasmann S. *et al.* Recruitment of entomopathogenic nematodes by insect–damaged maize roots. Nature 434, 732–737 (2005). 15815622 10.1038/nature03451

[b6] Kessler A. & Baldwin I. T. Defensive function of herbivore–induced plant volatile emissions in nature. Science 291, 2141–2144 (2001). 11251117 10.1126/science.291.5511.2141

[b7] Kappers I. F. *et al.* Genetic engineering of terpenoid metabolism attracts bodyguards to *Arabidopsis*. Science 309, 2070–2072 (2005). 16179482 10.1126/science.1116232

[b8] Heil M. *et al.* Evolutionary change from induced to constitutive expression of an indirect plant resistance. Nature 430, 205–208 (2004). 15241414 10.1038/nature02703

[b9] De Moraes C. M., Lewis W. J., Paré P. W., Alborn H. T. & Tumlinson J. H. Herbivore–infested plants selectively attract parasitoids. Nature 393, 570–73 (1998).

[b10] Weinkamer R. & Fratzl P. Mechanical adaptation of biological materials – the examples of bone and wood. Mater. Sci. Eng. C 31, 1164–1173 (2011).

[b11] Fratzl P. & Weinkamer R. Nature's hierarchical materials. Prog. Mater Sci. 52, 1263–1334 (2007).

[b12] Stokes A. & Berthier S. Irregular heartwood formation in *Pinus pinaster* Ait. is related to eccentric, radial, stem growth. Forest Ecol. Manag. 135, 115–121 (2000).

[b13] Telewski F. W. Is windswept tree growth negative thigmotropism? Plant Sci. 184, 20–28 (2012). 22284706 10.1016/j.plantsci.2011.12.001

[b14] Malik I. & Wistuba M. Dendrochronological methods for reconstructing mass movements – An example of landslide activity analysis using tree–ring eccentricity. Geochronometria 39, 180–196 (2012).

[b15] Berthier S., Kokutse A. D., Stokes A. & Fourcaud T. Irregular heartwood formation in maritime pine (*Pinus pinaster* Ait): consequences for biomechanical and hydraulic tree functioning. Ann. Bot. – London 87, 19–25 (2001).

[b16] Wardrop A. B. & Davies G. W. The nature of reaction wood. VIII. The structure and differentiation of compression wood. Aust. J. Bot. 12, 24–38 (1964).

[b17] Niklas K. J. & Spatz H. C. Wind–induced stresses in cherry trees: evidence against the hypothesis of constant stress levels. Trees 14, 230–237 (2000).

[b18] Jullien D., Widmann R., Loup C. & Thibaut B. Relationship between tree morphology and growth stress in mature European beech stands. Ann. Forest Sci. 70, 133–142 (2013).

[b19] Hamant O. *et al.* Developmental patterning by mechanical signals in *Arabidopsis*. Science 322, 1650–1655 (2008). 19074340 10.1126/science.1165594

[b20] Kierzkowski D. *et al.* Elastic domains regulate growth and organogenesis in the plant shoot apical meristem. Science 335, 1096–1099 (2012). 22383847 10.1126/science.1213100

[b21] Biechele T., Nutto L. & Becker G. Growth strain in Eucalyptus nitens at different stages of development. Silva Fenn. 43, 669–679 (2009).

[b22] Kübler H. Studies on growth stresses in trees, I: The origin of growth stresses and the stresses in transverse direction. Holz Roh. Werkst. 17, 1–9 (1959).

[b23] Han Z. W., Zhang J. Q., Ge C., Li W. & Ren L. Q. Erosion resistance of bionic functional surfaces inspired from desert scorpions. Langmuir 28, 2914–2921 (2012). 22208552 10.1021/la203942r

[b24] Han Z. W. *et al.* Anti-erosion function in animals and its biomimetic application. J. Bionic Eng. 7, S50–S58 (2010).

[b25] Ding L. X., Shi W. P. & Luo H. W. Numerical simulation of viscous flow over non-smooth surfaces. Comput. Math. Appl. 61, 3703–3710 (2011).

[b26] Song X. Q., Lin J. Z., Zhao J. F. & Shen T. Y. Research on reducing erosion by adding ribs on the wall in particulate two-phase flows. Wear 193, 1–7 (1996).

[b27] Finnie I. Some reflections on the past and future of erosion. Wear 186–187, 1–10 (1995).

[b28] Finnie I. Erosion of surfaces by solid particles. Wear 3, 87–103 (1960).

[b29] Alméras T., Thibaut A. & Gril J. Effect of circumferential heterogeneity of wood maturation strain, modulus of elasticity and radial growth on the regulation of stem orientation in trees. Trees 19, 457–467 (2005).

[b30] Yamashita S., Yoshida M., Takayama S. & Okuyama T. Stem–righting mechanism in gymnosperm trees deduced from limitations in compression wood development. Ann. Bot. 99, 487–493 (2007). 17218339 10.1093/aob/mcl270 PMC2802951

[b31] Beemster G. T. S. & Baskin T. I. Analysis of cell division and elongation underlying the developmental acceleration of root growth in *Arabidopsis thaliana*. Plant Physiol. 116, 1515–1526 (1998). 9536070 10.1104/pp.116.4.1515 PMC35060

[b32] Mattheck C. & Bethge K. The structural optimization of trees. Naturwissenschaften 85, 1–10 (1998).

[b33] Baskin T. Anisotropic expansion of the plant cell wall. Annu. Rev. Cell Dev. Biol. 21, 203–222 (2005). 16212493 10.1146/annurev.cellbio.20.082503.103053

[b34] Chen P. Y., McKittrick J. & Meyers M. A. Biological materials: functional adaptations and bioinspired designs. Prog. Mater Sci. 57, 1492–1704 (2012).

[b35] Wu D. Y., Meure S. & Solomon D. Self–healing polymeric materials: a review of recent developments. Prog. Polym. Sci. 33, 479–522 (2008).

[b36] Pouget E. *et al.* Hierarchical architectures by synergy between dynamical template self–assembly and biomineralization. Nat. Mater. 6, 434–439 (2007). 17515916 10.1038/nmat1912

[b37] Heuer A. H. *et al.* Innovative materials processing strategies: a biomimetic approach. Science 255, 1098–1105 (1992). 1546311 10.1126/science.1546311

[b38] Fratzl P. & Weiner S. Bio–inspired materials–mining the old literature for new ideas. Adv. Mater. 22, 4547–4550 (2010). 20878627 10.1002/adma.201002127

[b39] Zhu J. H. & Li J. S. A study on desertification of west Jilin province based on remote sensing and GIS techniques. Chinese Geogr. Sci. 12, 73–79 (2002).

[b40] Suresh S. & Giannakopoulos A. E. A new method for estimating residual stresses by instrumented sharp indentation. Acta Mater. 46, 5755–5767 (1998).

[b41] Xu Z. H. & Li X. D. [Residual stress determination using nanoindentation technique.]. Micro and nano mechanical testing of materials and devices. [Yang, F. & Li, J. C. M. (eds.)] [139–153] (Springer Science + Business Media, LLC, York, 2008).

[b42] Oliver W. C. & Pharr G. M. An improved technique for determining hardness and elastic modulus using load and displacement sensing indentation experiments. J. Mater. Res. 7, 1564–1583 (1992).

[b43] Oliver W. C. & Pharr G. M. Measurement of hardness and elastic modulus by instrumented indentation: Advances in understanding and refinements to methodology. J. Mater. Res. 19, 3–20 (2004).

[b44] Suresh S. & Giannakopoulos A. E. A new method for estimating residual stresses by instrumented sharp indentation. Acta Mater. 46, 5755–5767 (1998).

[b45] Khan M. K., Fitzpatrick M. E., Hainsworth S. V. & Edwards L. Effect of residual stress on the nanoindentation response of aerospace aluminium alloys. Comp. Mater. Sci. 50, 2967–2976 (2011).

